# RACIAL/ETHNIC DISPARITIES IN HELP-SEEKING BEHAVIORS IN OLDER AMERICANS WITH SUBJECTIVE COGNITIVE DECLINE

**DOI:** 10.1093/geroni/igad104.0481

**Published:** 2023-12-21

**Authors:** Xiang Qi, Yaolin Pei, Zheng Zhu, Bei Wu

**Affiliations:** New York University, New York City, New York, United States; New York University, New York City, New York, United States; New York University, New York City, New York, United States; New York University, New York City, New York, United States

## Abstract

Subjective cognitive decline (SCD) refers to individuals’ perceived decline in memory that negatively impacts the daily activities or work. Seeking help for SCD and/or related functional limitations may be beneficial as it can prompts screening and intervention for cognitive impairment. However, racial/ethnic minorities may face disadvantages in help-seeking behaviors due to healthcare access disparities, dementia-related stigma, and cultural differences. To investigate the racial/ethnic differences in help-seeking for cognitive problems among middle-aged and older adults with SCD and/or related functional limitation, we used data from the Behavioral Risk Factor Surveillance System (BRFSS) 2015-2021. The sample included 41,115 adults aged ≥45 with SCD and 20,227 (45.4%) with SCD-related functional limitation. We estimated the prevalence of help-seeking behaviors and conducted a logistic regression analysis accounting for BRFSS survey weights, missing data, state-level clustering, and covariates. Among those with SCD and/or related functional limitation, Asian American and Pacific Islanders (AAPIs) are least likely to initiate communication with their healthcare provider with their cognitive problems. In the multivariate logistic model, AAPIs (OR, 0.45; 95% CI, 0.33-0.61) and non-Hispanic blacks (OR, 0.76; 95% CI, 0.63-0.91) with SCD-related functional limitation were less likely to discuss cognitive problems compared to non-Hispanic whites. Our findings were consistent with studies suggesting that AAPIs with cognitive impairment were less likely to seek for dementia care due to their lower health literacy, limited English proficiency, and dementia-related stigma. Our study emphasizes the need for tailored interventions that promote early patient-provider communication to address racial/ethnic disparities in help-seeking for cognitive problems.

